# Mechanism of Endoplasmic Reticulum Stress in Cerebral Ischemia

**DOI:** 10.3389/fncel.2021.704334

**Published:** 2021-08-02

**Authors:** Yu Han, Mei Yuan, Yi-Sha Guo, Xin-Ya Shen, Zhen-Kun Gao, Xia Bi

**Affiliations:** ^1^Department of Sport Rehabilitation, Shanghai University of Sport, Shanghai, China; ^2^Department of Rehabilitation Medicine, Shanghai University of Medicine and Health Sciences Affiliated Zhoupu Hospital, Shanghai, China; ^3^Shanghai University of Medicine and Health Sciences Affiliated Shanghai University of Traditional Chinese Medicine, Shanghai, China

**Keywords:** cerebral ischemia, ER stress, unfolded protein response, apoptosis, inflammation, Ca^2+^ homeostasis

## Abstract

Endoplasmic reticulum (ER) is the main organelle for protein synthesis, trafficking and maintaining intracellular Ca^2+^ homeostasis. The stress response of ER results from the disruption of ER homeostasis in neurological disorders. Among these disorders, cerebral ischemia is a prevalent reason of death and disability in the world. ER stress stemed from ischemic injury initiates unfolded protein response (UPR) regarded as a protection mechanism. Important, disruption of Ca^2+^ homeostasis resulted from cytosolic Ca^2+^ overload and depletion of Ca^2+^ in the lumen of the ER could be a trigger of ER stress and the misfolded protein synthesis. Brain cells including neurons, glial cells and endothelial cells are involved in the complex pathophysiology of ischemic stroke. This is generally important for protein underfolding, but even more for cytosolic Ca^2+^ overload. Mild ER stress promotes cells to break away from danger signals and enter the adaptive procedure with the activation of pro-survival mechanism to rescue ischemic injury, while chronic ER stress generally serves as a detrimental role on nerve cells via triggering diverse pro-apoptotic mechanism. What’s more, the determination of some proteins in UPR during cerebral ischemia to cell fate may have two diametrically opposed results which involves in a specialized set of inflammatory and apoptotic signaling pathways. A reasonable understanding and exploration of the underlying molecular mechanism related to ER stress and cerebral ischemia is a prerequisite for a major breakthrough in stroke treatment in the future. This review focuses on recent findings of the ER stress as well as the progress research of mechanism in ischemic stroke prognosis provide a new treatment idea for recovery of cerebral ischemia.

## Introduction

Stroke is the second highest cause of death. Cerebral ischemia stemming from arterial occlusion is responsible for most of the stroke, a catastrophic illness leading to permanent disability in 80% of survivors (Moskowitz et al., 2010; Campbell et al., 2019; Jayaraj et al., 2019). In the face of such an intractable problem, relatively effective interventions to prevent death and improve brain cells recovery are in short supply today (Ekker et al., 2018; Zerna et al., 2018).

Endoplasmic reticulum served as a highly multifaceted organelle essentially differs from the surrounding cytoplasm both structurally and functionally (Sanderson et al., 2015). Scores of chaperones, enzymes, and cofactors associated with ER regulate precise folding of newly synthesized proteins and assist polypeptides in achieving final functional conformation (Chapman et al., 1998). Under the physiological environment, misfolded proteins are decreased and degraded by the accurate quality control mechanisms of proteasome, lysosome, and autophagy pathways to maintain the protein homeostasis called proteostasis in eukaryotic cells (Kaushik and Cuervo, 2015; Hetz and Saxena, 2017).

Apart from the dynamic integration of proteins, ER also plays an indispensable role in the intracellular Ca^2+^ homeostasis related to cell survival and neuronal plasticity (Mattson et al., 2000). The regulation of Ca^2+^ homeostasis by ER mainly depends on the dynamic balance of Ca^2+^ release and re-uptake in the lumen of the ER. Inositol 1,4,5-trisphosphate (IP3), a second messenger, mobilizes Ca^2+^ stored in ER directly and stimulates Ca^2+^ entry indirectly to regulate intracellular Ca^2+^ levels (Berridge and Irvine, 1989). IP3 receptor (IP3R) and ryanodine receptor (RyR) are part of Ca^2+^ release channels (Foskett et al., 2007; Van Petegem, 2012). Ca^2+^ pumps of sarcoplasmic/endoplasmic reticulum calcium ATPases (SERCA) are responsible for Ca^2+^ re-uptake (Takenaka et al., 1982). Most ER associated proteins are involved in maintaining ER Ca^2+^ homeostasis where Bip, GRP94, and cofactors are conducive to Ca^2+^ buffering in the ER lumen. Failure of the Ca^2+^ homeostasis by toxic stimuli such as ischemia, hypoxia, and hypertension (Marciniak and Ron, 2006; Walter and Ron, 2011) leads to ER stress which causes protein misfolding and generates a pathological state to cells in danger. When the accumulation of unfolded polypeptides or folding-incompetent proteins surpass the capacity of ER chaperones, a signal transduction pathway called UPR in ER lumen is preferentially activated ensure cells to restore homeostasis (Hetz, 2012; Hetz and Papa, 2018). However, a high level of insult above the regulation of UPR, sets a motion pro-apoptotic pathways making cells end up with death.

A variety of experimental studies have demonstrated that ER stress induced by cerebral ischemia could be a key element in complex pathology mechanisms involving neuronal cell, glial cells, and endothelial cells loss during or after cerebral ischemia (Rissanen et al., 2006; Zhao et al., 2018; Wang et al., 2019; Haupt et al., 2020). Most animal models used in experiments performed on transient cerebral ischemia models, which mimics the widespread clinical ischemia/reperfusion (I/R) where the occurrence of cortical spreading depression, periinfarct depolarization, and cerebral edema result in secondary neuronal damage and infarct expansion (Dohmen et al., 2008; Liebeskind et al., 2019). Sufficient evidence has suggested that targeted inhibition of ER stress and UPR can effectively improve experimental I/R injury. For instance, astragalin, a kind of flavonoid, significantly attenuated the expression levels of apoptotic proteins (Bax and cleaved-caspase-3) and the release of inflammatory cytokines, as well as the ER-related proteins, glucose-regulated protein to alleviate I/R injury via suppression of ER stress on rats after transient middle cerebral artery occlusion (MCAO). Similar results were obtained *in vitro* neuronal cell culture model (Liu et al., 2020). However, potential contamination, histological techniques, and methodological defects could be encountered in neuron cell culture technology (Molcanyi et al., 2013, 2014). Caution is required by interpreting any neuronal cell culture result. A recent study showed that acupuncture operated on Baihui (GV 20), and Qubin (GB7) acupoints in rats immediately after reperfusion can play a neuroprotection role to improve neurological scores and restrain autophagy and apoptosis induced by ER stress (Sun et al., 2020). Therefore, the mechanism of ER stress in cerebral ischemia is worth studying. This review aims to contribute to this growing area of research by summarizing the molecular pathways of ER stress controlling cell fate after cerebral ischemia.

## The UPR of ER Stress Signaling in Cerebral Ischemia

**FIGURE 1:**
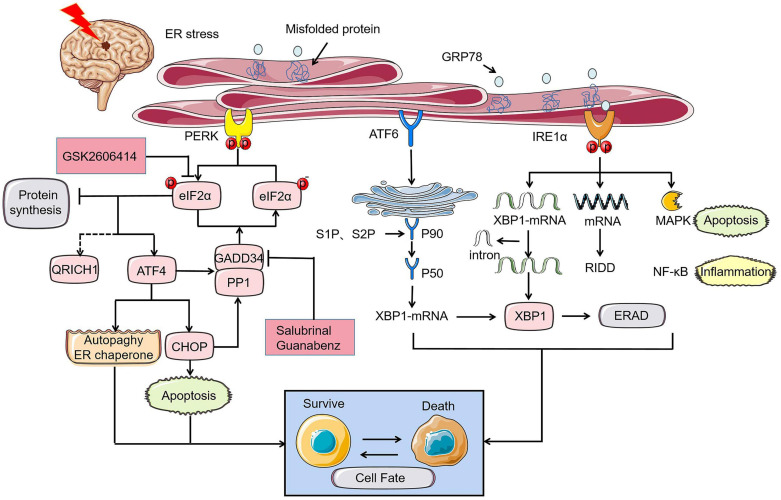
UPR of ER stress in cerebral ischemia determines cell fate through PERK, IRE1, and ATF6 pathways. Under the ischemic state, PERK-eIF2α-ATF4-CHOP axis controls cell fate by apoptosis where p- eyIF2α play a key role in inhibiting protein synthesis and its role will be reversed by PERK inhibitor GSK2606414. GADD34, which is simultaneously regulated by ATF4 and CHOP promotes promoting the dephosphorylation of eIF2α under prolonged ER stress. Similarly, the role of GADD34 can disappear with the intervention of salubrinal and guanabenz. The rescue of cell fate by ATF4 is mainly achieved by inhibiting autophagy and controlling the ER chaperones. The different expression of XBP1 induced by ATF6 and IRE1α can also bring disparate endings to the cell. MAPK apoptosis pathway and NF-κB inflammation related to IRE1α can directly determine cell survival.

The UPR served as stress sensors and regulators of downstream transcription factors monitors unfolded and misfolded proteins aggregation and recode genes to determine cell destiny (Chen and Brandizzi, 2013; Chow et al., 2015). This phenomenon is the main subsequent performance after ER stress. Initiation of UPR depends on the activation of three type-I transmembrane proteins namely protein kinase RNA-like ER kinase (PERK), inositol-requiring protein 1 (IRE1), and activating transcription factor-6 (ATF6). Under the condition of neuronal homeostasis, these three proteins are in devitalized state binding to glucose regulated protein 78 (GRP78), a major molecular ER chaperone. GRP78 separated from PERK, IRE1, and ATF6 bind to misfolded proteins upon ER dysfunction in pathological state caused by ischemic stroke, indicating UPR initiation. Phosphorylation of eukaryotic initiation factor 2α (eIF2α) induced by PERK inhibit protein synthesis to reduce the load of unfolded proteins on ER and upgrade the expression of ER chaperone to rectify misfolded proteins and restore folding order (DeGracia and Montie, 2004). Newly synthesized proteins ultimately identified as nonfunctional will be reversely transported from ER to cytosol. The process by which the substrates are subsequently polyubiquitinated and degraded by proteases is known as ER-associated degradation (ERAD) (Lopata et al., 2020). Typically, activated IRE1 and cleaved ATF6 involve in ERAD induced by X-box binding protein 1 (XBP1) (Chen and Brandizzi, 2013; Sun et al., 2015; Jung et al., 2019; Waldherr et al., 2019). Within a few hours after I/R, PERK and IRE1 will be activated to varying degrees. Various damage mechanisms related to cerebral ischemia control the fate of nerve cells by altering the expression of UPR induced mRNA (Figure 1; Deng et al., 2004). UPR down-regulates protein translation and up-regulates ER-related enzymes, cofactors, and chaperones alleviating ER stress to play a neuroprotective role in ischemic injury. During intense ER stress and prolonged UPR in cerebral ischemia, the early adaptive protection of UPR to cells could be replaced to cell programmed death.

### PERK Pathway

#### PERK and eIF2α

Protein kinase RNA-like ER kinase, a type I transmembrane ER-resident protein, is a central part of UPR, as its signaling pathway regulates the control of protein synthesis and translation to a large extent. The occurrence of PERK activation suppresses the level of global protein synthesis momently through targeting phosphorylation eIF2α at serine residue 51 when misfolded and unfolded protein accumulates under the ER stress after transient global brain ischemia (Harding et al., 1999; Owen et al., 2005; Marciniak et al., 2006). eIF2 is essential for the initiation of mRNA translation (Kimball, 1999). Therefore, phosphorylation of eIF2α has been regarded as a specific mechanism to inhibit translation and decrease the protein load of ER. It skillfully links the upstream kinase of eIF2α with the downstream translation events.

The preservation of protein synthesis inhibition (PSI) is closely related to neuronal survival following brain ischemia and reperfusion. The phosphorylation of eIF2α, which appears in the short-lived acute stage of brain ischemia, is responsible for the first few hours of PSI and does not explain the continued prolongation of PSI. Recently, researchers found that Hes1 knockdown aggravated the neural apoptosis through the PERK pathway where the level of p-eIF2α enhanced dramatically in transient cerebral ischemia model because the increase of infarct size and a deterioration of the nervous system are counteracted with PERK-specific inhibitors GSK2606414 (Li Y. et al., 2020). In another experiment, PERK-specific knockout mice induced by hybridization and tamoxifen showed larger infarct volume and worse neurological function scores with low expression of p-eIF2α in 3-day and 3-week recovery after MCAO compared to control mice (Wang et al., 2020). This indicates that the lack of PERK-induced phosphorylation of eIF2α and subsequent inhibition of translation is an important factor leading to neurological damage in cerebral ischemia. Therefore, inhibition of protein synthesis is the main way for PERK to restore neurological function after cerebral ischemia. Interestingly, the knock-down and activation of PERK by different means both have aggravated the functional impairment after ischemia, which may associates with the duration of UPR. In the future, the targeted intervention of PERK could be a potential therapeutic direction for stroke.

#### ATF4

Although PERK-mediated phosphorylation of eIF2α inhibits global protein translation, it specifically upregulates the translation of some mRNAs including the activating transcription factor 4 (ATF4) due to ATF4 mRNA open reading frames (ORF2) existing specific sequence (Harding et al., 2000). The expression level of ATF4 mRNA has been verified to be enhanced by anoxia in primary rat fibroblasts in Estes et al. (1995). ATF4 diverges into two branches. ATF4 is usually described as a transcriptional activator controlling the fate of cells under stress, which has received widespread attention in cerebral ischemic injury. Recently, the research has indicated that overexpression of ATF4 induced by adeno-associated virus protects against I/R injury, reduces the infarct volume and neurological scores with suppression of inflammatory response (He et al., 2019).

Activation of ATF4 downstream target transcription factors and association with other signaling pathways predict cellular adaptive regulation or death program involved in pro-apoptosis, autophagy, and protein synthesis. In terms of pro-survival mechanisms, the expression of various adaptive genes including ER chaperones, metabolic enzymes, AA transporters is promoted by ATF4 to relieve stress and fit cells into an adaptive environment. The brain tissue of animals in hibernation can mimic a variety of stress response states including ischemia, hypoxia, oxidative stress. in brain tissue from hibernating squirrels, ATF4 was found to transfer to the nucleus and form a compound with the nucleus co-factor CREB1 to which combined with the promoter region of GRP78 to support cell survive (Mamady and Storey, 2008). On the contrary, C/EBP homologous protein (CHOP), a pro-apoptotic factor is directly induced by ATF4 during ER stress guiding cell death (Harding et al., 2002; Marciniak et al., 2004). In the investigation of the neuroprotective mechanism of granulocyte colony stimulating factor (G-CSF)on ischemic stroke, it was found that the expression of ATF4, CHOP, caspase-12 related to the apoptosis pathway induced by ER stress decreased, and the mice showed signs of better ER homeostasis reconstruction and less acute neuronal degeneration of post-ischemic stroke (Modi et al., 2020).

Activating transcription factor 4 also indirectly interacts with autophagy and protein synthesis related pathways. In terms of autophagy regulation, ATF4 silencing attenuates the expression of PARK2, weakens the phagocytosis induced by PARK2, and reverses the neuroprotective effects of ER stress activators tunicamycin and thapsigargin in the *vivo* and *vitro* model of ischemia (Zhang X. et al., 2014). Protein synthesis regulation induced by ATF4 is accompanied through enhancing the translation of the growth-arrest-and DNA-damage-induced transcript 34 (GADD34) which dephosphorylates eIF2α to recover protein synthesis (Marciniak et al., 2004).

Whether autophagy or protein synthesis, once the regulatory intensity exceeds the threshold of suitable living context, it will lead to tragedy. Only maintenance of intracellular protein homeostasis and effective establishment of recycling of cellular material can save cells from the stress response.

#### CHOP

C/EBP homologous protein, a key transcription factor downstream of perk- eIF2α-ATF4, plays an important role in apoptosis induced by ER stress. The expression of CHOP is at a low level under physiological conditions and elevates dramatically when cells are stimulated by stress. CHOP mRNA induction was observed in the rats’ hippocampus suffering from global cerebral ischemia (Paschen et al., 1998). CHOP is a pro-apoptotic factor. It cannot directly cause apoptosis but mediates apoptosis by regulating the expression of downstream apoptosis-related factors BCL-2 and caspase-12 (Oida et al., 2008). According to studies, CHOP promotes cell death still by increasing protein synthesis, which leads to oxidative stress (Marciniak et al., 2004; Han et al., 2013). The up-regulation of CHOP due to I/R with the TUNEL-positive neuron cells, which were co-located with CHOP and caspase-12, decayed (Zhao et al., 2018) is counteracted by the intervention of chrysophanol. Decrease of CHOP expression and increase of ER oxidoreductin-α (Ero1-α) induced by hypothermic reversed apoptosis in the models of ischemic stroke compared to normal temperature (Poone et al., 2015). CHOP is detected in the nuclei of damaged neurons after 24 h of bilateral common carotid artery occlusion. *In vitro* experiment, the primary hippocampal neurons of wild-type mice are more sensitive to apoptosis induced by oxygen and glucose deprivation (OGD)/reoxygenation compared with CHOP^–/–^mice (Tajiri et al., 2004). These all suggest that knock-down and inactivation of CHOP facilitate the nerve cells’ recovery in ER stress due to cerebral ischemia. Moreover, CHOP controls cell death, depends on the intensity of the stress. Only under intense stress, CHOP strongly dominates apoptosis factors and thus induces cell death, but under mild stress, it cannot add the intensity of stress and remarkably promote protein secretion with the result of low correlation with apoptosis (Southwood et al., 2002). It is worth noting that recently, p-eIF2α raises the translation of QRICH1 which accelerates the rate of cell death during ER stress in intestinal epithelial cells and also involves the regulation of transcription procedures that promote protein secretion. It can be inferred that QRICH1 may be a new transcription regulator on the PERK-eIF2α axis. However, the effects and location of QRICH1 require to be confirmed in cerebral ischemia (You et al., 2021).

#### GADD34

Under ER stress, ATF4, CHOP, and a great deal of mRNA of encoding transcription factors together induce the expression of GADD34, which bound to the serine/threonine protein phosphatase 1 (PP1) is an integral component for eIF2α phosphatase complex. There has been controversy about the decision of GADD34 on cell fate in cerebral ischemia. In early studies, it was found that GADD34 was up-regulated in neurons and microglia in the peri-infarct region of cerebral ischemia rodents and the human brain after cardiac arrest (Imai et al., 2002; White et al., 2004). The increase in GADD34 expression can restore protein synthesis inhibition and participate in DNA repair to promote cell survival. Unexpectedly, The cerebral ischemia model mice injected with the viral vector containing the full-length GADD34 showed more severe infarction volume than no insert full-length GADD34 (McCabe et al., 2008). Restoring protein synthesis by dephosphorylation of eIF2α is an important step in reversing damage, but at the same time, it will enhance the accumulation of misfolded proteins and cause further damage. The molecular mechanism of mammalian cells restoring normal protein synthesis after ER stress associated with ischemia is explained by the role of GADD34:PP1 complex dephosphorylation. The experiment has demonstrated that inactivation of GADD34 decreased the damage during ER stress without excessive protein accumulation (Marciniak et al., 2004).

Salubrinal, an eIF2α dephosphorylation inhibitor, is equivalent to reach the p-eIF2α state in disguise by dissociating GADD34:PP1 complex to preserve nutrition and lessen ER chaperones to neutralize damage of ER stress (Boyce et al., 2005). The reduction of CA1 cell death and blood-brain barrier damage is found in the salubrinal-treated mice model of global cerebral ischemia (Nakka et al., 2010; Anuncibay-Soto et al., 2016). However, some studies have found that the intervention of salubrinal can aggravate neuronal apoptosis and degeneration *in vivo* and *in vitro*, and exacerbate neurobehavioral damage in intracerebral hemorrhage models (Meng et al., 2018). Furthermore, there is a standpoint that salubrinal is regarded as an inhibitor of the eIF2α phosphatases which lacks an experimental basis (Boyce et al., 2005). The production of the paradox and contradictory experimental results may attribute to the dissimilar time node and duration of eIF2α phosphorylation under different stress levels. The specific evidence-based basis needs further scientific exploration. However, guanabenz complex selectively disrupting dephosphorylation of eIF2α by binding to GADD34:PP1 complex regulates the rate of protein synthesis within the controllable range of the ER chaperones to protect cells from the pressure of protein misfolding (Tsaytler et al., 2011). Phosphorylation of eIF2α as a hub to alleviate ER stress will become a heated issue in the treatment of neurological diseases in the future.

### IRE1 Pathway

#### IRE1

IRE1α, a most conserved transducer of UPR, distributes abundantly in various tissues in response to ER stress and contains a serine/threonine kinase domain and an endoribonuclease domain (Tirasophon et al., 1998; Kim et al., 2008). Similar to PERK, GRP78 dissociates from IRE1α and approaches to dimerization and autophosphorylation of IRE1 under ER stress. Subsequently, the endonuclease activity of IRE1 specifically splices the mRNA encoding the transcription factor X-box binding protein 1 (XBP1) to remove a 26-nucleotide intron from the coding region of XBP1 mRNA (Calfon et al., 2002). Translocation of XBP1 mRNA open reading frame prompt it to be translated into XBP1s protein, an active transcription factor upregulating pro-survival signaling (Paschen et al., 2003). XBP1s specifically induces the expression of target genes involved in ERAD and protein folding and has completed related events that control protein homeostasis to unload ER lumen (Hetz et al., 2015).

The result that the increased translation of GRP78, GRP94, and other glucose regulatory proteins due to XBP1s bind to unfolded protein contributes to the recovery of ER stress (Lee A.-H. et al., 2003). In the *in vitro* model of I/R, overexpression of XBP1 induced by adenovirus suppressed cell death induced by OGD/R stress, relieving the damnification of ER stress (Ibuki et al., 2012). Icariin enhanced the cell viability of primary cortical neurons and weakened inflammation levels after OGD/R injury via restraining the IRE1α-XBP1 signaling pathway where the expression of XBP1s, IL-β, IL-6, TNF-α, the ratio of p-IRE1α/IRE1αare decreased (Mo Z.-T. et al., 2020). Melatonin protected more neurons survival in mice with cerebral ischemia (Lin et al., 2018) via inhibiting the PERK pathway. Interestingly, Melatonin treatment before ischemia can inhibit endoplasmic reticulum stress-dependent autophagy through the PERK and IRE1 pathways to relieve cerebral edema and neuronal apoptosis (Feng et al., 2017).

After the mitigation of ER stress, similar to the principle of eIF2α dephosphorylation, spliced XBP1 mRNA is gradually replaced by an unspliced form. A great deal of mRNA and micro RNAs regulated by the activated IRE1α plays a role in multiple signaling pathways, mediating inflammation, apoptosis, lipid synthesis, and protein secretion, which is known as IRE1α-dependent mRNA decay (RIDD) (Hollien and Weissman, 2006). However, the research of RIDD in ischemic diseases is relatively few. In addition, activated IRE1 recruits binding protein associated with stress pathways, including mitogen-activated protein kinase (MAPK) pathway inducing apoptosis. Inflammation pathway mainly governed by nuclear factor-κB (NF-κB), and these cascade events have been validated in cerebral ischemia (Borsello et al., 2003; Hu et al., 2006; Luo et al., 2008; Zhang et al., 2016). In short, as a branch of UPR, IRE1 participates in the mechanism program of various cell survival and determines cell fate under cerebral ischemia.

### ATF6 Pathway

#### ATF6

Activating transcription factor-6 is another type-I transmembrane protein in ER lumen besides PERK and IRE1. ATF6 disassociated from GRP78 is translocated to Golgi apparatus where inactive 90 kDa ATF6 cleaved by endopeptidase S1P and S2P is converted into activated 50 kDa ATF6 and then transported to nucleus regulating gene expression (Ye et al., 2000). ATF6 also has two cell control mechanisms, pro-apoptosis, and pro-survival. ATF6 cooperates with IRE1 to control the translation of XBP1 and improves the ERAD by inducing α-mannosidase-like protein 1 (EDEM1) and GRP78 (Yamamoto et al., 2007). Not only in cerebral ischemia models, but also myocardial ischemia, renal ischemia, and other ischemic models, the protective effect appears in the cell homeostasis of the pharmacological ATF6 reprogramming protein induced by a compound called 147 and disappears after the absence of specific ATF6. In this experiment, the expression of GRP78 and catalase which recently proved to be a principal members of the novel antioxidant gene program induced by ATF6 were both elevated (Blackwood et al., 2019). Treatment strategy based on protein homeostasis induced by ATF6 may restore the injury of insecure proteostasis cerebral ischemia (Thiebaut et al., 2019). Moreover, a study indicates that ATF6 is the chief protein that enhances neuroprotection within 2 h before I/R (Urban et al., 2009). On the contrary, the role of taurine in alleviating ER stress is to inhibit ATF6, which is considered to be a detrimental factor in this experiment in MCAO and neuron culture models (Gharibani et al., 2013). The result may be attributed to the fact that ATF6 is also present in the promoter of gene encoding CHOP in addition to ATF4 and XBP1. The clear nature of ATF6 is still controversial. Cleaved ATF6 is a powerful marker of UPR activation. Unfortunately, there are fewer studies on the ATF6 branch in the ER stress after cerebral ischemia than the PERK and IRE1 arm given its difficult to detect. All the above-mentioned studies related to UPR pathway have been summarized (Table 1).

**TABLE 1:** Studies on intervention of UPR in cerebral ischemia.

UPR	Intervention	Related protein changes	Ischemia model	Effects	References
PERK pathway	Hes1 knockdown	Activating the PERK/eIF2α/ATF4/CHOP signaling pathway	tMCAO	Knockdown of Hes1 increased cerebral infarction, worsened nervous system prognosis, and promoted ER stress-induced apoptosis.	Li L. et al., 2020
	PERK-cKO by cross-breeding Camk2a-CreERT2 with *Perkf/f* mice	Decreasing the expression of p-eIF2α and p-perk	tMCAO/BCAO	Decreased neurological scores and increased infarct volume in PERK-cKO mice.	Wang et al., 2020
	Overexpression of ATF4 induced by adeno-associated virus	Increasing the expression of ATF4	MCAO	Overexpressed ATF4 reduced cerebral infarction volume, lowered neurological score and improves HE and Nissl staining.	He et al., 2019
	Giving mice a subcutaneous injection of G-CSF (50 μg/kg) for 4 days	Decreasing the expression of ATF4, CHOP, capase-12	BCAO	G-CSF can’t only reduce acute neuronal degeneration, but also increase long-term plasticity after cerebral ischemia, and maintain cell homeostasis by reducing pro-apoptotic proteins and increasing anti-apoptotic proteins.	Modi et al., 2020
	ATF4 silencing	Decreasing the expression of PARK2 and PARK2-dependent mitophagy	tMCAO	The silencing of ATF4 gave rise to the disappearance of the neuroprotective effects where the volume of cerebral infarction decreases and the absorption rate of glucose in ischemic tissue increases induced by tunicamycin and thapsigargin.	Zhang X. et al., 2014
	Continuous injection of chrysophanol (0.1 mg/kg) for 14 days	Decreasing the expression of CHOP, GRP78, p-eIF2α, caspase-12 and increasing the expression of anti-inflammatory factor IκB-α	MCAO	CHR exerted anti-inflammatory effects by inhibiting ER stress response after I/R while reducing neuronal apoptosis.	Zhao et al., 2018
	Hypothermia (31°C)	Decreasing the expression of CHOP and Ero1-α	MCAO	Hypothermia inhibited apoptosis of stroke cells induced by endoplasmic reticulum stress.	Poone et al., 2015
	Salubrinal	Increasing the expression of p-eIF2α and GADD34	MCAO	Salubrinal played a neuroprotective effect by reducing CA1 cell death and blood-brain barrier damage.	Nakka et al., 2010; Anuncibay-Soto et al., 2016
IRE1 pathway	Overexpression of XBP1s induced by adenovirus transduction	Increasing the expression of XBP1s	ODG/R of primary rat hippocampal neurons	Overexpression of XBP1s suppressed cell death induced by OGD/R stress.	Ibuki et al., 2012
	Give Icariin to microglia and hippocampal neurons 1 h before OGD	Decreasing the ratio of p-IRE1α/IRE1α, the expression of XBP1s and IL-β, IL-6, TNF-α	ODG/R of primary microglia and cortical neurons	Increased viability of primary cortical neuron cells treated with Icariin.	Shaulian and Karin, 2002
	Using melatonin (5 mg/kg) at the beginning of reperfusion	Suppressing the PERK/eIF2α/ATF4/CHOP signaling pathway	MCAO	The infarct volume and individual skin lesion size of melatonin-treated mices were significantly reduced, and the number of surviving neurons increased.	Lin et al., 2018
	Melatonin pretreatment before cerebral ischemia	Decreasing the expression of p-perk and p-IRE1	tMCAO	Melatonin pretreatment markly relieved cerebral infarction, cerebral edema, neuronal apoptosis and nervous system defects.	Feng et al., 2017
ATF6 pathway	ATF6 pharmacological activation by a compound called 147	Increasing the expression of GRP78 and catalase	MCAO	The pharmacological activation of ATF6 stabilized the reprogramming protein, reduces damage and preserves brain function.	Blackwood et al., 2019
	Taurine	Downregulating the ratio of cleaved ATF6, full-length ATF6, p-IRE1, caspase-12, CHOP and Bax	MCAO ODG/R of primary cortical neurons	Taurine not only caused neuroprotection through the ATF6 and IRE1 pathways, but also reduces apoptosis and cerebral infarction volume in these model.	Gharibani et al., 2013

## Ca^2+^ Homeostasis and ER Stress in Cerebral Ischemia

### Ca^2+^ Homeostasis in the ER

Ca^2+^ homeostasis makes a difference in integrating multiple brain functions in the nerve cells across the cytomembrane and between the cytoplasm and intracellular organelles (Zündorf and Reiser, 2011). Under normal physiological conditions, ER lumen provides a high Ca^2+^ place where the concentration is higher than cytoplasm for ER-client enzymes and chaperones to maintain ER functions with an efficient operation (Mattson et al., 2000; Michalak et al., 2002; Coe and Michalak, 2009) and involve in rapid Ca^2+^ signaling events associated with chemical and electrical cell stimulation.

As mentioned earlier, Ca^2+^ homeostasis in the ER benefits from the dynamic balance of Ca^2+^ release and re-uptake. The generation of IP3 is a secondary event induced by the agonist-dependent hydrolysis of the lipid (Berridge, 1989). The stimulation of phospholipase C hydrolyzing the membrane lipid phosphatidylinositol 4,5-bisphosphate to gain IP3, which is the follow-up reaction of cell surface receptors activation due to extracellular agonists (Furuichi et al., 1989). IP3Rs are expressed in all cell types and active during above-mentioned state. RyRs, another calcium release channel, are mainly distributed in neurons and skeletal muscle, which discharge Ca^2+^ from ER lumen back into cytoplasm (Foskett et al., 2007; Gong et al., 2019) to cut down the concentration within ER together with IP3Rs (Santulli et al., 2017). In parallel, SERCA pumps Ca^2+^ from the cytoplasm into ER lumen to recovery of Ca^2+^ concentration in the ER. A recent study showed that SERCA2, a P-type ion-motive ATPase that resides on the ER membrane, is regarded as a gatekeeper of neuronal calcium homeostasis in the central nervous system (CNS) (Britzolaki et al., 2018).

In addition to the source of cytoplasm, Ca^2+^ supplement also involves extracellular pathways. ER Ca^2+^ from the extracellular environment through the plasma membrane (PM)cannot do without the store operated Ca^2+^ entry (SOCE) along with the activation of neuronal SOC channels (Chang, 2006; Smyth et al., 2010; Mohammed Thangameeran et al., 2020). The conception of SOCE dates back to Putney (1986) but the exact regulatory mechanism is unclear. SOCE is executed by the stromal interaction molecule 1 and 2 (STIM1/2), which act as Ca^2+^ sensors located in ER membrane and plasma membrane protein ORAI1, which function as pore-forming subunits of SOCE channels (Smyth et al., 2010). After depletion of ER Ca^2+^ induced by IP3R due to physiological extracellular stimuli, Ca^2+^ separates oneself from the STIM1/2 N-terminal domain, which leads to STIM1/2 undergoing complex conformational changes and oligomerization, allowing oligomer to activate and bine ORAI1 in ER-PM junctions. The best understood of SOCE is the Ca^2+^ release-activated Ca^2+^ (CRAC) channel trapped in the ER-PM junctions in response to depletion of ER Ca^2+^ to trigger Ca^2+^ influx (Lewis, 2020).

### Disruption of Ca^2+^ Homeostasis in Cerebral Ischemia

Both dysregulation of Ca^2+^ homeostasis in the ER and Ca^2+^ overload in cytoplasm plays a key role in cell apoptosis under an ischemic state. Ca^2+^ imbalance and ER stress are inseparable where various chaperones, enzymes, and cofactors cannot assist in synthesizing protein normally due to depletion of Ca^2+^ in the ER. The interruption of energy supply due to oxygen and glucose deprivation following cerebral ischemia can give rise to the deficiency of ATP ion channels and sustained neuronal depolarization. Under the state of abnormal electrochemical gradient and cells depolarization, over-opening voltage-dependent Ca^2+^ channels (VDCCs) and unintentional release of the neurotoxic excitatory neurotransmitter glutamate which activates N-methyl-d-aspartate receptors (NMDARs) leading to incremental intracellular Ca^2+^ concentration and excitotoxicity (Szydlowska and Tymianski, 2010; Singh et al., 2019). Neurons and oligodendrocytes are intensively sensitive to NMDA mediated excitotoxicity, which is responsible for their fatal death at the early stage.

Although astrocytes and endothelial cells have a longer survival period in ODG, they eventually die after reoxygenation due to energy compensation mechanism where the compensatory hypoxic protective response of astrocyte system can provide sufficient oxygen supply and energy substrate for neurons (Lee D.R. et al., 2003).

Ca^2+^ overload involves several initiations of biochemical and physiological reactions and actives Ca^2+^-associated proteins, which include phospholipases, endonucleases, caspase, calpains, and other kinases causing a series of neuronal cell death (Singh et al., 2019). One of the typical events of the following excitotoxicity after cerebral ischemia is pathologic activation of calpains. The cleavage of neuronal substrates via calpain adversely affects the structure and function of neurons. Various proteins located in membrane (L-type calcium channels, PMCA, and NCX) and ER (IP3R, RyR, and SERCA) involving in Ca^2+^ homeostasis have been discovered to be cleaved by calpain with its greater activation *in vivo* or *in vitro* under the excitotoxicity (Vosler et al., 2008). For example, calpain inhibits IP3 metabolism to make it act on IP3R for a longer time, which undergirds Ca^2+^ efflux from ER (Bevers and Neumar, 2008). An intervention to calpain inhibition has been proved to be a useful treatment for I/R. Calpeptin and MDL28170 termed calpain inhibitors reduced neuroinflammation and neuronal apoptosis after focal cerebral I/R injury in rats (Peng et al., 2011; Wang et al., 2021).

To make matters worse, the initiation of SOCE engenders Ca^2+^ influx in response to the overstimulation of glutamate receptors (Berna-Erro et al., 2009). As thus double whammy of Ca^2+^ inflow from the activation of NMDARs and SOCE may raise the concentration of Ca^2+^ to a critical level in which brain cells are extremely prone to death (Serwach and Gruszczynska-Biegala, 2019). Various components of SOCE playing a key role in cerebral ischemia have been shown under extensive researches. For capacitive Ca^2+^ entry (CCE) and Ca^2+^ overload induced by ischemia, the regulating effect of STIM2 is more significant than STIM1 in neuronal hypoxic cell death (Berna-Erro et al., 2009). Compared with wild-type mice, hippocampal neurons from STIM2 KO mice have a higher survival rate and lower level of ER Ca^2+^ concentration under hypoxic space both in culture and in acute hippocampal slices (Berna-Erro et al., 2009). Exhaustion of STIM2 is better protected against ischemic damage obviously (Berna-Erro et al., 2009). Even so, the importance of STIM1 cannot be overlooked in terms of improving neurological function. Intracellular high Ca^2+^ concentration due to the increased STIM1 and ORAI1 levels that occur in global ischemia rats dropped significantly with the injection of STIM1 siRNA, and then the rats had fewer neurological deficits compared with control (Zhang M. et al., 2014). ORAI1 is essential for pathological thrombus formation. Analogously, mice with deletion of ORAI1 were resistant to ischemic brain infarction (Braun et al., 2009). Those findings show that pharmacologic intervention of SORE to reduce or treat the formation of thrombus may be a potential therapeutic strategy to manage cerebrovascular events (Mammadova-Bach et al., 2019).

### ER–Mitochondria Crosstalk of Ca^2+^ in Cerebral Ischemia

Apart from ER, Ca^2+^ regulation of mitochondria in the occurrence and development of cerebral ischemia is indispensable when it comes to ion homeostasis. The close connection between ER and mitochondria is indicated by the uptake of Ca^2+^ from the ER to mitochondria with the aid of mitochondria-associated ER membranes (MAMs). Mitochondria, an organelle that sustains energy in living organisms, can be transformed into cell killers under massive harmful stimuli. Generally, mitochondria and ER interact to form a closely related whole that controls cell fate through the regulation of Ca^2+^ signaling pathways.

In cerebral ischemia, mitochondrial dysfunction caused by mitochondrial oxidative stress leads to neuronal death, and Ca^2+^ imbalance is the primary factor in this result. Extreme intracellular Ca^2+^ release from ER lumen and activation of NMDA following ischemia cause Ca^2+^ overload in mitochondrial matrix. Subsequently, excessive Ca^2+^ entering the mitochondria opens the mitochondrial permeability transition pore (MPTP), which induces the release of cytochrome c and initiates cell apoptotic program by activating caspases-3 and caspases-9 (Celsi et al., 2009). Cyclophilin D (CypD) recognized as a regulator of MPTP has been targeted by most MPTP inhibitors (Briston et al., 2019). Significant reduction in brain infarct size and neuroprotection shown in CypD-deficient mice after acute middle cerebral artery occlusion and reperfusion indicated that CypD plays a key role in ischemic models referring to Ca^2+^ overload and oxidative stress (Schinzel et al., 2005). Moreover, ER Ca^2+^ release and mitochondrial Ca^2+^ uptake are prevented with the participation of c- Jun N- terminal kinase1/2 (JNK1/2) small siRNA, which suggests JNK also contributes to the opening of MPTP and cell death (Verma et al., 2013). Therefore, the effective control of Ca^2+^ homeostasis to restrict the opening of MPTP may become the direction of efforts to reduce neuronal death.

Besides Ca^2+^, reactive oxygen species (ROS) generation is also a critical mechanism in ER–mitochondria crosstalk to regulate cell death (Marchi et al., 2018). On the one hand, high intracellular Ca^2+^ concentration augments the synthesis of nitric-oxide (NO), which enhances the production of ROS via disturbing the electron transport of mitochondria (Moncada and Erusalimsky, 2002). SERCA pump is presented at weak activity by the formation of NO and ischemic absence of ATP hydrolysis (Parsons et al., 1999). RyR2 is overactivated due to the excessive ROS in ischemia (Bull et al., 2008), which contributes to the higher intracellular Ca^2+^ concentration. On the other hand, mitochondrial Ca^2+^ is combined with cardiolipin to disintegrate respiratory chain complex II resulting in ROS generation and oxidative stress (Hwang et al., 2014). Superfluous ROS will bring about accumulation of oxidatively modified and misfolded proteins in the ER, which destroys ER homeostasis and causes ER stress (Brunet et al., 2000). In brain cells, astrocytes have better resistance to ROS, while endothelial cells are vulnerabal to ROS (Ouyang et al., 2007; Hertz, 2008).

### Ca^2+^ Homeostasis and ER Stress

Destruction of Ca^2+^ homeostasis due to the absence of energy in ischemia state will lead to ER stress which operates UPR to eliminate the accumulation of misfolded protein in the ER. Regulation of UPR is related to Ca^2+^ to a certain degree. For example, Calreticulin, an ER resident Ca^2+^ buffer can respond to Ca^2+^ depletion to stop assisting the combination of GRP78 and ATF6 to activate ATF6 (Hong et al., 2004). Not only abnormal activation of SOCE but also PDIA6, an ER resident oxidoreductase interacting on the domain of IRE1α will enhance IRE1α activity under ER stress. It is vital to show that the activity of PERK pathway will not be impacted when IRE1α activity is promoted by PDAI6 (Groenendyk et al., 2014). Those outcomes suggest that the activation of UPR branches is related to distinct Ca^2+^ regulation components and keeps relatively independent. Nowadays, thapsigargin and tunicamycin have been widely used as ER stress agonists in experiments by inhibiting SERCA and protein glycosylation, respectively (Zhang X. et al., 2014). In summary, starting from the mechanism of Ca^2+^ homeostasis destroyed by cerebral ischemia and restoring it could inhibit ER stress and save cell life.

## ER Stress and Apoptosis

**FIGURE 2:**
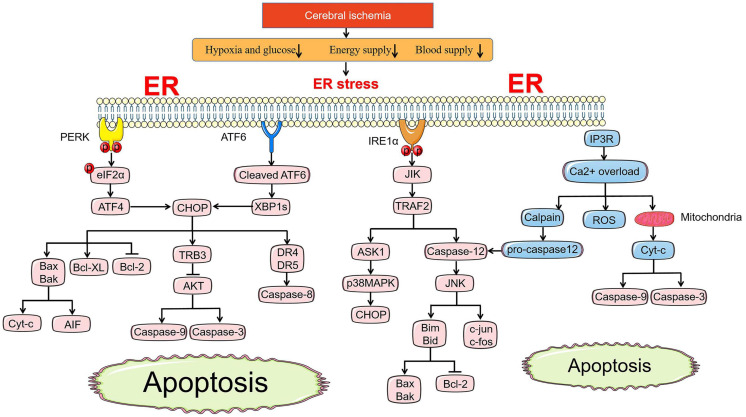
ER stress-mediated apoptosis pathway under cerebral ischemia. Under ischemic state, PERK and ATF6 both involve in apoptosis pathways induced by CHOP. BCL family, TRB3-AKT axis and death receptor DR4/5 are all regulated by CHOP to promote the release of Cyc-c and AIF, active the caspase-9, caspase-3, and caspase-8, respectively. JIK-TRAF2 apoptosis pathway induced by IRE1α and Ca^2+^ overload promote the activation of caspase-12, which accelerates the pro-apopotic protein of BCL family by controlling JNK. Ca^2+^ overload leads to the release of ROS directly and Cyt-c indirectly activating caspase-9 and caspase-3 via cross-talk with mitochondria.

The pathways of apoptosis induced by ER stress are different from those of mitochondria and death receptors, but they are connected in tandem to form a whole apoptotic network. CHOP, ASK1-JNK, and Caspase-12 pathway activation are recognized as three main apoptosis pathways under ER stress (Figure 2; Kaufman, 2002).

Protein kinase RNA-like ER kinase, IRE1, and ATF6 both induce the transcription of CHOP leading to cell apoptosis with prolonged ER stress. To a certain extent, the current researches are only relevant to the upstream regulation mechanism of CHOP associated with apoptosis and the downstream regulation mechanism is still vague. The expression of the TRB3 gene is upregulated by CHOP inhibiting the phosphorylation of Akt, a crucial anti-apoptotic signaling molecule, which successively activates the caspase-9 and caspase-3 (Datta et al., 1997; Ohoka et al., 2005). The level of Bcl2-family proteins is regulated by CHOP to induce apoptosis in ER stress. CHOP can upregulate the pro-apoptotic gene Bax and Bak, which enhances the release of cytochrome c (Cyt-C) and apoptosis inducing factor (AIF) involving in the mitochondrial apoptosis pathway and can suppress the expression of anti-apoptotic protein Bcl-2 and Bcl-XL promoting apoptosis (Fu et al., 2010; Tuzlak et al., 2016). Furthermore, CHOP mediates apoptosis in tandem with the death receptor pathway where the caspase-8-mediated cascade event happens after the elevated death receptor 4 (DR4) and DR5 due to the regulation of CHOP (Lu et al., 2014). The downstream apoptotic pathway of CHOP is not limited to ER stress alone, and more attention could be paid to the overall apoptosis formed by its cross-talk with mitochondrial pathways and death receptors in exploring the treatment of cerebral ischemia.

Apoptosis signal-regulating kinase 1-JNK pathway is another apoptotic pathway in ER stress where tumor necrosis factor receptor associated factor 2 (TRAF2) is deemed as a mainstay. First of all, under physiological conditions, TRAF2 combines with procaspase-12 to form a stable complex, while under ER stress, IRE1 activates C-jun-N-terminal-inhibiting kinase (JIK) to phosphorylate TRAF2, which separates procaspase-12 from TRAF2 and activates caspase-12 via homodimerization, causing cell apoptosis (Noguchi et al., 1999; Yoneda et al., 2001). On the other hand, the JIK-IRE1 complex recruits TRAF2 and leads to apoptosis signal-regulating kinase 1 (ASK1) activation acting on the downstream JNK and p38 mitogen activated protein kinases (p38MAPK) which phosphorylation modifies CHOP to regulate the apoptosis (Hatai et al., 2000). Pharmacological regulation of p38MAPK can reduce cell injury and apoptosis under ER stress after I/R (Li L. et al., 2020). Activated JNK is translocated to the nucleus to phosphorylate c-jun and c-fos, and then induces the expression of downstream apoptotic genes and ligand proteins such as FASL and TNF to initiate apoptosis pathway of death receptor via a transcription dependent manner (Shin et al., 1998). The role of c-Jun in the onset of apoptosis death in neurons has been demonstrated in cerebral ischemia (Xiao et al., 2018). Moreover, similar to the CHOP pathway in I/R models, JNK also upregulates the expression of pro-apoptotic BH3-only proteins Bim and Bid which inhibits the activity of Bcl-2 anti-apoptotic members or activates Bax/Bak-like pro-apoptotic members to mediate the mitochondrial pathway of apoptosis (Putcha et al., 2003; Guan et al., 2006).

Caspases-12 is located in the outer membrane of the ER and is only activated under ER stress. It is a key molecule that mediates ER stress and apoptosis. Calpain associated with mitochondrial dysfunction is activated owing to excessive Ca^2+^ in the cytoplasm, which cleaves the procaspase-12 located in ER and assists in the release activated caspase-12 into the cytoplasm (Nakagawa and Yuan, 2000; Chan et al., 2002). The above mentioned TRAF2 is directly related to the activation of caspase-12. A study has shown that the intervention of JNK inhibitors in rats after MCAO reduces not only the expression of p-JNK but also the caspase-12, which means JNK may be involved in the activation of caspase-12 (Zhu et al., 2012). According to the report, caspase-7 moved to the surface of the ER can cleave procaspase-12 of pro-domain to engender activated caspase-12 (Rao et al., 2001). Caspase-7 mutants and the addition of caspase-7 inhibitor in hippocampal cultured neurons with glucose deprivation significantly decline the expression of caspase-12 (de la Cadena et al., 2014). Finally, caspase-12 is specifically activated and activates caspase-9 in collaboration with other ER stress molecules, which then cause fine apoptosis through caspase-3. In addition to apoptosis induced by caspase-12, a research found that caspase-4 can be seen in humans as ER stress-specific caspase and may be involved in the pathogenesis of Alzheimer’s disease (Hitomi et al., 2004). MCAO rats treated by Tanshinone IIA (TSA)showed a reduction in infarction area, accompanied by a decrease in caspase-3 and caspase-8 compared with the control (Zhou et al., 2015). The relationship between the caspase family and apoptosis is quite consanguineous and complex. It is necessary to clarify the roles of initiator and effector in the upstream and downstream of death signal transduction.

## ER Stress and Inflammation

**FIGURE 3:**
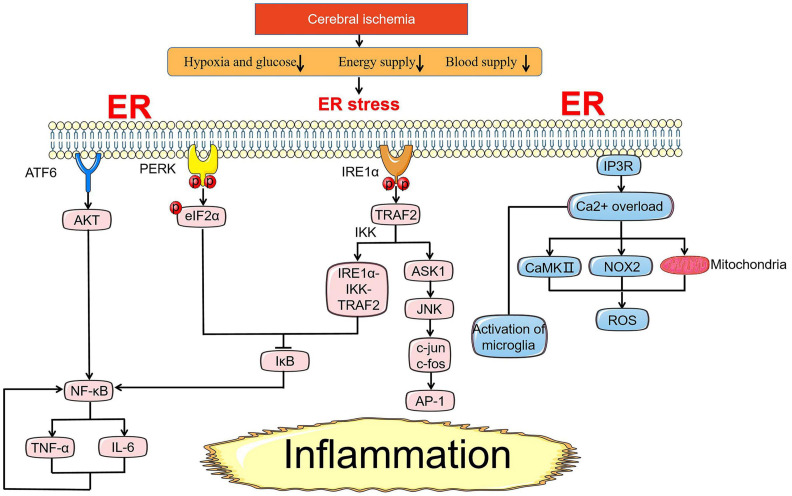
ER stress-mediated inflammation pathway under cerebral ischemia. Under ischemic state, NF-κB is an essential pivot in the inflammation pathway induced by ER stress. NF-κB is controlled by the activation of AKT and suppression of inhibitory IκB proteins. NF-κB and TNF-α promote each other casing nausea cycle of inflammation response. In addition to NF-κB, the release of inflammatory factors induced by IRE1α-TRAF2-JNK axis is also a crux part of the injury mechanism in cerebral ischemia. Ca^2+^ overload from the ER through NOX2-dependent, subsequent activation of CaMKII and mitochondria pathway lead to the generation of ROS involving in inflammation response. Reactive microglia are mainly expressed as M1 pro-inflammatory type with Ca^2+^ overload after I/R injury.

In addition to regulating the apoptosis pathway by coordinating ER stress-related genes to control cell survival, UPR seeks to inform cells in a dangerous context through an inflammatory pathway that is significant for the innate immune response to alleviate ER stress (Figure 3). Similarly, the qualitative release of inflammatory signaling depends on the intensity of stress where inflammatory signaling assists in relieve cell injury under transient ER stress, while prolonged stress will directly link inflammation with cell death. Inflammatory signaling pathways in cerebral ischemia include toll-like receptors (TLR) signaling pathway, MAPK signaling pathway, and NF-κB signaling pathway, the latter two are mainly driven by UPR under ER stress (Marsh et al., 2009; Lanzillotta et al., 2013; Mo Y. et al., 2020).

After cerebral ischemia, the NF-κB inflammatory pathway is most relevant to ER stress, which precisely stems from the cell-autonomous pro-inflammatory transcription programs mediated by the three arms of the UPR all gathering to the generation of pro-inflammatory factors with the location of NF-κB in nucleus. Under cells homeostasis state, the incorporation of NF-κB and several inhibitory IκB proteins isolates inactivated NF-κB in the cytoplasm (Gilmore, 2006). With initiation of UPR under ER stress, the decreased expression of IκB and activated NF-κB is detected in cells as PERK phosphorylates eIF2α reducing the overall protein synthesis (Deng et al., 2004). This suggests that eIF2α phosphorylation excites NF-κB by suppressing the level of IκB where the mechanism of activation is ambiguous. Some scholars inferred that the occurrence of this phenomenon may be the increased proportion of NF-κB to IκB promoting more NF-κB transfer to the nucleus due to a shorter half-life of IκB compared with NF-κB (Sprenkle et al., 2017). The active IRE1α recruits NF-κB kinase inhibitors IκB kinase (IKK) near the ER to form an IRE1α-IKK-TRAF2 complex which causes phosphorylation and ubiquitination of IκB. Consequently, IκB is degraded by protease leading to NF-κB dissociation and translocation to the nucleus, which induces the production of pro-inflammatory cytokine TNF - α and IL-6 accelerating stress-induced cell death (Hu et al., 2006). In addition, the existence of cleaved ATF6 also makes sense in the activation of NF-κB. Transient phosphorylation of AKT was discovered before subtilase cytotoxin (SubAB) activated NF-κB and among the three signaling pathways of UPR, only gene and pharmacological inhibition of ATF6 can reduce the expression of p-AKT and the activation of NF-κB (Yamazaki et al., 2009). ROS as a core mediator of inflammatory response is responsible for the stimulation of NF-κB by ER stress (Pahl and Baeuerle, 1996). The source of ROS in the body is excessive Ca^2+^ in the cytoplasm. High Ca^2+^ concentration in mitochondria results in mitochondrial dysfunction releasing ROS abnormally (Malhotra et al., 2008). On the other hand, Ca^2+^ efflux from the ER through NOX2-dependent and subsequent activation of calcium dependent protein kinases (CaMKII) lead to the generation of ROS (Li et al., 2010). In tMCAO models, treatment of cerebral ischemia with Xyloketal B (Xyl-B) can dramatically reduce the excessive production of ROS in brain tissue, suppress the expression of NF-κB and inducible nitric oxide synthase (iNOS), and down-regulate the mRNA levels of pro-inflammatory cytokines, including IL-1β, TNF-α, IL-6, and IFN-γ (Pan et al., 2017). Substantial evidence indicates that anti-inflammatory therapy focusing on NF-κB after cerebral ischemia requires focusing on the regulation of UPR and Ca^2+^ homeostasis.

In MAPK signaling pathway, JNK pathway induced by IRE1α provides a manner by which not only apoptosis signaling, inflammatory signaling is also free. AP-1, a heterodimer composed of c-Fos and c-Jun, deemed as an activating transcription in cells is stimulated to accelerate the transcription of pro-inflammatory cytokines after activation of JNK regulated by ASK1 (Tang et al., 2001; Shaulian and Karin, 2002). Furthermore, the release of p38 MAPK due to ASK1 also acts directly on inflammation mediated by cerebral damage (Sun and Nan, 2016). Robenacoxib, an anti-inflammation drug, has been demonstrated to have a deleterious effect that accelerates neuronal death. Interestingly, the side effects of using robenacoxib are offset with the combination of ER stress inhibitor salubrinal and robenacoxib to treat cerebral ischemia. The neuroprotective effect is reflected in the decreased activation of glial cells, especially microglia, which reduces the degeneration and loss of neurons caused by inflammation (Anuncibay-Soto et al., 2018). This indicates that ER stress and microglia may have an unknown connection to control inflammation.

In some non-ischemic models, other inflammatory pathways related to ER stress have been found. It has been shown that the reciprocity of nucleotide-binding oligomerization domain 1 and 2 (NOD1/2) and serine/threonine protein kinase 2 (RIPK2) could be enhanced by IRE-1α-TRAF2 axis leading to the activation of NF-κB under ER stress induced by brucella abortus infection (Keestra-Gounder et al., 2016). A research actualized via animal and clinical trials suggested that conjugated bile acids (CBAs) mainly combine with ATF6α alleviating airway inflammation with the attenuation of the IL-4, IL-5, IL-13 production (Nakada et al., 2019).

Actually, the conduction of inflammatory signaling from ER stress is bidirectional rather than a one-way street. The release of inflammatory mediators such as IL-6 and TNF-α will also accelerate the activation of UPR and release more inflammatory factors to produce a nausea cycle of inflammatory response where the mechanism involved may be related to the cascade events caused by overload Ca^2^ including mitochondrial dysfunction and accumulation of misfolded proteins in ER lumen (Xue et al., 2005).

## ER Stress and Glial Cells and Endothelial Cells in Cerebral Ischemia

As mentioned earlier, the involvement of neuronal cells in ER stress after cerebral ischemia is concernful certainly. However, the risk of stroke depends not only on the neuron in the brain but also on the function and health of endothelial and glial cells in general.

The consequences of early ischemic injury to endothelial cells may be wider scope than indicated by the small volume they occupy. Subtle changes are discovered in microvessels after ischemic injury. Adhesion molecules and regulatory cytokines located at the membranes of endothelial cells have interaction with activating leukocytes, which activates platelets and promotes micro thrombosis, thereby aggrandizing the infarct range in the ischemic area (Garcia et al., 1994). Clinically, brain edema after cerebral ischemia is a common cause of death. Ischemic stroke with poor prognosis largely depends on the formation of severe edema. The damage caused by increased intracranial pressure is irreversible (Liebeskind et al., 2019). Endothelial cells are the main component of whole blood-brain barrier (BBB). During cerebral ischemia, endothelial cells contracted, endothelial space expanded, BBB permeability increased, resulting in vasogenic brain edema (Petito et al., 1982). In addition to endothelial cell swelling, the increase of the terminal volume of astrocytes surrounding endothelial cells may potentially compromise the BBB function. Subsequently, the leakage of BBB will contribute to the fortified blood protein extravasation and further aggravate the angiogenic edema. With the aid of a reasonable amount of lithium, the damaged endothelial cells are repaired, which enhances the vasodilation ability and contribution to the stabilization of BBB and alleviation of edema (Bosche et al., 2016a,b; Haupt et al., 2020). Mechanisms underlying brain swelling are not clear but Ca^2+^ overload in endothelial cells has been identified as the main factor (Olson et al., 1990). The augment of Ca^2+^ concentration in endothelial cells is mainly due to the release of ER Ca^2+^ channel, which implies that endothelial cells could have a certain inevitable connection with ER stress. Fortunately, despite the small number of relevant studies, some have shown that endothelial cells are directly involved in the complex pathological mechanism of ER stress induced by cerebral ischemia. Pretreatment with siRNA-vascular endothelial growth factor (VEGF) before OGD/R changed the biological characteristics of brain-derived Endothelial cells 3 (BEND3), decreasing the expression level of XBP1, CHOP, and GRP78 (Feng et al., 2019). Astragaloside IV can effectively reduce the permeability of BBB by inhibiting the apoptosis of endothelial cells mediated by ER stress after I/R (Hou et al., 2020). Those researches indicate that endothelial cells are certainly correlated with ER stress. However, more sufficient experiments and arguments are needed to clarify the regulatory mechanism.

Glial cells are divided into astrocytes, oligodendrocytes, and microglia cells, which are activated in different degrees in response to cerebral ischemia. After 3 days of cerebral ischemia, GRP78 expression was highly correlated with the activation of microglia around infarction and the activation of astrocytes in the peri-infarct area (Jin et al., 2018). As just noted, the interaction between ER stress and inflammation is regulated by phenotype of reactive microglia. Astrocytes and pyramidal neurons in CA1 region emerged ER stress dependent apoptosis after ischemia. Notably, ameliorated CHOP in neurons remained unchanged in astrocytes (Osada et al., 2010). Similarly, neurovascular unit presents a structure-dependent response to ischemia with the salubrinal treatment. Descendent GFAP, a marker of activated astrocytes were detected in CA1 but no change in CA3. Outcomes of this research also revealed that endothelial cells are highly responsive to salubrinal, while astrocytes have limitations to responsiveness (Anuncibay-Soto et al., 2016). Therefore, decreased intensity of ER stress after cerebral ischemia hinges on differential responses state from different neurovascular units. Oligodendrocytes are sensitive to excitotoxicity and ER stress induced by ischemia and eventually produce oxidative stress and apoptosis. Regretfully, whether oligodendrocytes are involved in the regulation of ER stress and its specific mechanisms need to be further explored (Ruiz et al., 2020).

The concept of the neurovascular unit takes the integrity of brain function and the interaction between different structures into account, which provides a new mentality for clinical treatment of ischemic stroke.

## Conclusion and Perspectives

The evidence for activation of pathways associated with ER stress after cerebral ischemia is strong and the life of brain cells cannot be separated from the UPR propagated or induced by ER stress. Various enzymes and transcription factors downstream of UPR will control cell fate according to the intensity and duration of ER stress. However, there is still controversy regarding the increased or decreased expression of transcription factors such as ATF4, GADD34, XBP1s, ATF6. downstream of UPR in the neuroprotective effect of cerebral ischemia. Therefore, the critical point and clear molecular mechanism of UPR’s reversal need to be further discussed. Therefore, breakthrough research on related small molecule inhibitors under ER stress will be what stroke patients and neurologists are eager to see.

Although the positive effect of small molecule inhibitors of targeted ER stress in experimental cerebral ischemia has been proven, such as dantrolene (Li et al., 2005), salubrinal, 4-PBA, and guanabenz, the successful verification of these in clinical trials is rarely reported. The ER is closely related to calcium ions, and the most urgent affair to alleviate ER stress after cerebral ischemia is to restore Ca^2+^ homeostasis. It is a good idea to save cell fate from the apoptosis pathway and inflammation pathway related to ER stress. The treatment and mechanism of cerebral ischemia of different cell groups should be taken into consideration rather than limited to neurons. What remains to come up with is a complete and relatively mature response plan to deal with nerve damage after cerebral ischemia.

A better understanding of the diversiform mechanisms involved in ER stress will contribute to identify useful cerebral ischemia treatments. Further studies ought to address the unknown downstream molecular mechanisms, the potential for digging deep into known pathways of ER stress response and their implications in neurological diseases beside cerebral ischemia.

## Author Contributions

MY and Y-SG designed the structure of the manuscript. X-YS and Z-KG managed the literature searches and analyses. YH wrote the manuscript. XB assisted with the improvement of the manuscript. All authors contributed to and have approved the final manuscript.

## Conflict of Interest

The authors declare that the research was conducted in the absence of any commercial or financial relationships that could be construed as a potential conflict of interest.

## Publisher’s Note

All claims expressed in this article are solely those of the authors and do not necessarily represent those of their affiliated organizations, or those of the publisher, the editors and the reviewers. Any product that may be evaluated in this article, or claim that may be made by its manufacturer, is not guaranteed or endorsed by the publisher.
